# Changes in Clinical Practice Reduce the Rate of Anastomotic Leakage After Colorectal Resections

**DOI:** 10.1007/s00268-017-4423-7

**Published:** 2017-12-28

**Authors:** Henrik Iversen, Madelene Ahlberg, Marja Lindqvist, Christian Buchli

**Affiliations:** 1 Department of Molecular Medicine and Surgery, Karolinska Institutet and Center for Digestive Diseases, Karolinska University Hospital, Solna, P9:03, 17176 Stockholm, Sweden; 2 Department of Physiology and Pharmacology, Karolinska Institutet and Department of Anesthesiology, Surgical Services and Intensive Care Medicine, Karolinska University Hospital, Stockholm, Sweden

## Abstract

**Background:**

Anastomotic leakage is a serious clinical problem after colorectal resections and is associated with a significantly increased length of stay, morbidity and mortality. The aim of the present study was to evaluate the effect of changes in clinical practice on anastomotic leakage rate after colorectal resections.

**Methods:**

Retrospective cohort study based on prospectively collected data. All 894 patients with primary anastomosis after colorectal resection at a tertiary referral center between 2006 and 2013 were analyzed. Changes in clinical practice aiming at reducing the rate of anastomotic leakages were introduced in January 2010 and were characterized by exclusion of perioperative nonsteroidal anti-inflammatory drugs, introduction of intra-operative goal-directed fluid therapy and avoidance of primary anastomoses in emergency resections. The study population was divided into two groups, one treated before and one after the introduction of changes in clinical practice. Groups were compared regarding patient characteristics and incidence of anastomotic leakage.

**Results:**

The cumulative incidence of anastomotic leakage after colorectal resections decreased from 10.0% (41 of 409) to 4.5% (22 of 485) after changing clinical practice, relative risk 0.45 (95% CI 0.27–0.75, *p* = 0.002). The adjusted odds ratio was 0.45 (0.26–0.78, *p* = 0.004). A separate analysis showed a decrease after colon resections from 9.1% (23 of 252) to 4.5% (14 of 310), relative risk 0.49 (0.26–0.94, *p* = 0.039), and from 11.5% (18 of 157) to 4.6% (8 of 175) after rectal resections, relative risk 0.40 (0.18–0.89, *p* = 0.024).

**Conclusion:**

Implementing a structured change of clinical practice can significantly reduce the anastomotic leakage rate after colorectal resections.

**Trial registration:**

Clinical trial registration number: ACTRN12617001497392.

## Introduction

Anastomotic leakage after colorectal surgery is a serious complication which is associated with severe morbidity, increased length of stay and overall mortality [1–4]. The reported incidence of anastomotic leakage varies between 1.8 and 19%, typically higher in low rectal anastomoses and in randomized or population-based studies [2, 4–6].

Several risk factors for anastomotic leakage are not adjustable, and the potentially adjustable factors are difficult to affect by the surgeon [2, 3, 7].

In 2009 the first reports were published indicating that nonsteroidal anti-inflammatory drugs (NSAIDs) may have a detrimental effect on colorectal anastomotic healing in patients [8]. Currently, the effect of NSAIDs on anastomotic healing has not been assessed in a large randomized controlled trial. A recent meta-analysis, however, suggests caution when prescribing NSAIDs to patients with preexisting risk factors for anastomotic leakage [9].

Intra-operative goal-directed fluid therapy improves outcomes in major surgery and has been shown to reduce postoperative morbidity and length of stay [10–12].

Emergency colon resection has been identified as an independent risk factor for anastomotic leakages, and defunctioning stoma reduces the incidence of clinically relevant anastomotic leakages after low anterior resection [2, 5, 13].

Surgical skills and techniques affect oncological outcomes for patients operated for colorectal cancer [14]. The principles of total mesorectal excision (TME) according to Heald were introduced at our institution in 1992, and complete mesocolic excision (CME) according to Hohenberger has been consistently applied since 2004 [15, 16]. A standardized enhanced recovery program (ERP) was implemented in 2006 [17]. These measures improved oncological outcomes and decreased the length of stay. However, we considered the incidence of anastomotic leakage after colorectal resections being unsatisfactory high. In an attempt to reduce the leakage rate after colorectal resections changes in clinical practice were introduced at the Section for Coloproctology at the Karolinska University Hospital in 2010.

The hypothesis was that exclusion of perioperative NSAIDs, introduction of intra-operative goal-directed fluid therapy and avoidance of primary anastomoses in emergency resections would reduce the incidence of anastomotic leakage.

The aim of this cohort study was to assess the combined effect of these changes in clinical practice on the incidence of anastomotic leakage.

## Methods

### Study design, setting and participants

This is a cohort study that included all consecutive patients treated with colon or rectal resection and primary anastomosis at the Karolinska University Hospital, a tertiary referral center, between January 2006 and December 2013. Colorectal resections were performed with standardized mainly open surgery during the study. Surgical principles, preoperative and postoperative care remained unchanged during the entire study period. Structured changes in clinical practice aiming at reducing the anastomotic leakage rate were introduced in January 2010. Cumulative incidence of anastomotic leakage was the primary outcome and compared between patients treated before (period 1: 2006–2009) and after (period 2: 2010–2013) implementing the changes in clinical practice.

### Changes in clinical practice

Changes in clinical practice aiming at reducing the risk of anastomotic leakage included three key elements:

1. NSAIDs were not allowed from 5 days before surgery to 7 days after surgery. For patients with a history of ischemic heart disease low dose (≤75 mg daily) acetylsalicylic acid was, however accepted, irrespective of EDA. Oral slow release opioids in combination with naloxone replaced the NSAIDs.
2. Goal-directed intra-operative fluid therapy was managed by stroke volume (SV) optimization using esophageal Doppler (CardioQ-ODM™; Deltex Medical, Chichester, UK). Fluid loading was done using 200 ml of 6% hydroxyethyl starch solution or a dextran 60 solution. If SV increased ≥10%, the fluid challenge was considered positive and an additional fluid bolus was given. After SV optimization was achieved, the epidural was activated. During the course of surgery, optimization maneuvers were repeated at the discretion of the anaesthetist.
3. Surgeons were instructed to avoid emergency oncological resections of obstructing cancers by instead performing temporary defunctioning ostomies. Primary anastomosis was not recommended if an emergency resection was unavoidable (i.e., bowel perforation, ischemia or severe bleeding).

### Data source and definition of anastomotic leakage

The same research nurse recorded data prospectively throughout the whole study period on sex, age, diagnosis, surgical procedure, length of stay, elective/emergency surgery, type of complications (including anastomotic leakage) and surgical re-interventions in a dedicated database. Computed tomography with rectal contrast enema was the standard examination in patients with a deviant postoperative course and was liberally performed. Occurrence of anastomotic leakage was registered during index hospital stay or 30 days postoperatively. Visible signs of an anastomotic defect during reoperation, leakage of luminal contrast on radiological assessment or need for percutaneous drainage of any intraabdominal fluid collection near the anastomotic site was regarded as anastomotic leakage. Anastomotic leakage was thus defined according to the International Study Group of Rectal Cancer as a defect of the intestinal wall at the anastomotic site (including suture and staple lines of neorectal reservoirs) leading to a communication between the intra- and extraluminal compartments, or as an abscess adjacent to the anastomosis [18]. Early postoperative radiology with rectal contrast enema was not routinely performed for all patients. This was only performed in case of a deviant postoperative course, e.g., leukocytosis or fever. Hence, only grade B and C leakages requiring any therapeutic intervention were included in this trial.

### Surgical techniques

Anterior resection implied TME surgery with transection of the inferior mesenteric artery (IMA) [19, 20] with ligation of the inferior mesenteric vein and the left colic artery at the same level. The marginal artery and terminal bifurcation of the ascending left colic artery were spared in order to preserve adequate blood supply to the descending colon in case of an insufficient marginal artery at the splenic flexure [21]. The left colon was divided after complete mobilization of the splenic flexure in order to resect the sigmoid together with the specimen. The rationale for a complete resection of the sigmoid colon was to obtain a better anastomotic blood perfusion, since dividing the IMA results in a decreased arterial perfusion in the sigmoid colon which may decrease tissue oxygenation in the proximal limb and hence increase the risk of anastomotic leakage [22–24]. Before transecting the colon the marginal artery and vasa recta supplying the proximal staple line were indentified and preserved. A stapled side-to-end colorectal anastomosis was performed [25, 26]. Except for non-irradiated women without other risk factors a defunctioning loop ileostomy was recommended. In sigmoid resections the IMA was divided at its origin, the bowel transected at the level of the promontory and the anastomosis was stapled. In right-sided colectomies the ileocolic vessels were divided at their origin together with the right branch of the middle colic artery after identification of the superior mesenteric vein. Anastomoses were hand sewn or stapled. The splenic flexure was resected and the middle colic artery divided at its origin in extended right-sided colectomies or resections of the transverse colon.

There were no changes in operative techniques in period 2 compared to period 1.

### Management of anastomotic leakage

Symptomatic anastomotic leakages with no sign of fecal peritonitis were managed by transanal or percutaneous drainage and antibiotics. If there were signs of fecal peritonitis a laparotomy was performed. In severe cases the anastomosis was taken down and a descending colostomy created. In cases of limited pelvic sepsis with only a minor anastomotic defect a protective ileostomy was created if not already present. Patients with anastomotic leakage after colonic resections were managed similarly, but the anastomosis was more likely taken down with a creation of a deviating ostomy. However, if bowel continuity was present and sepsis well confined to an abscess adjacent to the anastomosis the patient could be managed more conservatively with percutaneous drainage and antibiotics. The management of anastomotic leakage was the same during the whole study period.

### Enhanced recovery protocol

All electively treated study participants were included in the ERP, implemented in January 2006. The ERP included preoperative carbohydrate-rich beverage, intra-operative buffered glucose solution (25 mg ml^−1^) at a rate of 2–3 ml kg^−1^ h^−1^ to replace insensible loss and norepinephrine to achieve a mean arterial pressure of above 60–70 mm Hg. Oral bowel preparation was restricted to patients scheduled for anterior resection. For analgesia, a low thoracic epidural catheter was inserted before induction of anesthesia and celecoxib, a selective cyclooxygenase (COX) 2 inhibitor, was routinely used for postoperative pain management.

### Statistics

Study data were recorded in a dedicated database and analyzed by Stata version 12 (StataCorp LP, College Station, TX, USA). Groups were compared with nonparametric tests and Fisher’s exact tests. Univariable logistic regression was used to assess the crude effect of study period and other predictors on anastomotic leakage. Potential confounding and/or effect modulation of age, sex, type of resection (colonic versus rectal), type of pathology (diagnosis), simultaneous (multivisceral) resections, acute or elective surgery and protective ostomy was assessed with multivariable logistic regression analysis. Covariates that changed the unadjusted odds ratio (OR) by more than 10% were regarded as important predictors and included in the final model to report adjusted OR. Interaction terms with a *p* value below 0.05 were regarded as significant.

## Results

### Participants

The reason for surgery did not change between the two time periods and were colorectal neoplasia in 85%, inflammatory bowel disease (IBD) in 13% and other pathology in 2%. Age, sex, type of resection, the proportion of multivisceral resections and protective ostomies were similar in both groups (Table 1). The proportion of emergency resections decreased significantly from 14 to 4% (*p* < 0.01) in colon resections and from 4% to zero (*p* = 0.01) in rectal resections (Table 1).

**Table 1:** Characteristics of patients with primary anastomosis after colorectal resections

Variable	Period 1 (*n* = 409)	Period 2 (*n* = 485)	*p* value
Sex ratio (M:F)	234:175	262:223	0.35
Age* (years)	66 (26–89)	65 (17–97)	0.54
Type of pathology			0.69
Cancer	347 (85)	412 (85)	
IBD	55 (13)	61 (13)	
Other	7 (2)	12 (2)	
Type of resection			0.49
Colon	252 (62)	310 (64)	
Rectal	157 (38)	175 (36)	
Multivisceral resection	128 (31)	163 (34)	0.47
Protective ostomy			
Colon	21 (8)	26 (8)	1.00
Rectum	116 (74)	137 (78)	0.39
Emergency resection			
Colon	36 (14)	11 (4)	<0.01
Rectum	6 (4)	0 (0)	0.01

Period 1 (2006–2009) *versus* period 2 (2010–2013), after introduction of changes in clinical practice aiming at reducing anastomotic leakages

Values in parentheses are percentages. *p* value Fisher’s exact. *Wilcoxon rank-sum test

### Cumulative incidence of anastomotic leakage

The cumulative incidence of anastomotic leakage was 10.0% (41 of 409) before the change in clinical practice (period 1) and decreased to 4.5% (22 of 485) after the change in clinical practice (period 2) with a relative risk (RR) for anastomotic leakage of 0.45 (95% CI 0.27–0.75, *p* = 0.002). For colon resections the cumulative incidence decreased from 9.1% (23 of 252) to 4.5% (14 of 310) and for rectal resections from 11.5% (18 of 157) to 4.6% (8 of 175). The relative risk was 0.49 (95% CI 0.26–0.94, *p* = 0.039) for colon and 0.40 (95% CI 0.18–0.89, *p* = 0.024) for rectal resections, respectively (Table 2).

**Table 2:** Cumulative incidence of anastomotic leakage after colorectal resections with primary anastomosis

Type of resection	Period 1		Period 2			*p* value
	*n*	Leakage	*n*	Leakage	RR (CI)	
Colon and rectum	409	41 (10.0)	485	22 (4.5)	0.45 (0.27–0.75)	0.002
Colon	252	23 (9.1)	310	14 (4.5)	0.49 (0.26–0.94)	0.039
Rectum	157	18 (11.5)	175	8 (4.6)	0.40 (0.18–0.89)	0.024

Period 1 (2006–2009) *versus* period 2 (2010–2013), after changing clinical practice aiming at reducing anastomotic leakages

Values in parentheses are percentages. *RR* relative risk, *CI* confidence interval. *p* value Fisher’s exact

**Fig. 1:**
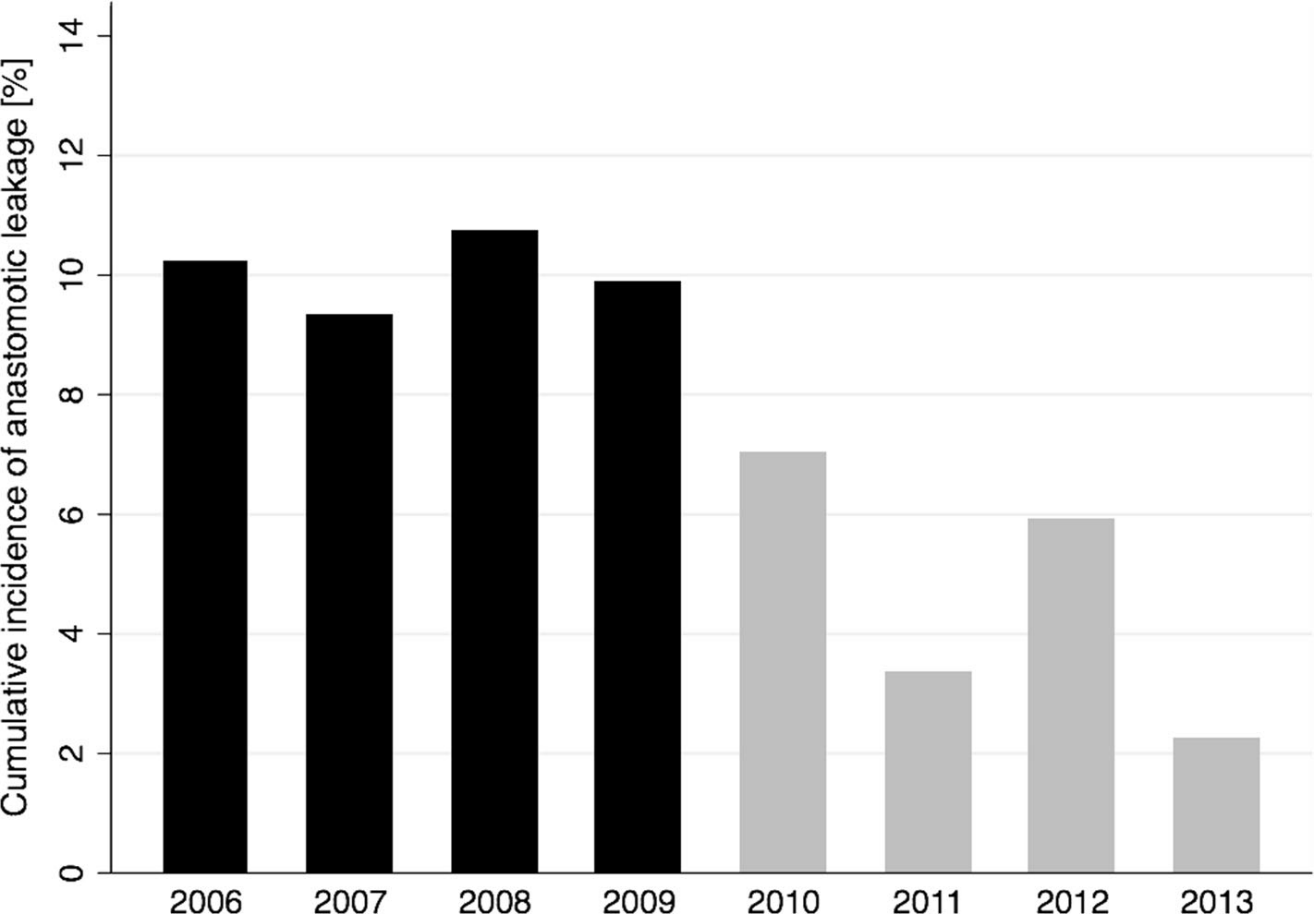
Cumulative incidence of anastomotic leakage per year for patients with primary anastomosis after colorectal resections. Changes in clinical practice aiming at reducing anastomotic leakages were introduced in 2010

The effect of the changes in clinical practice was similar in the analysis restricted to elective colorectal resections with RR 0.47 (95% CI 0.28–0.79, *p* = 0.004). Figure 1 displays the cumulative incidence of anastomotic leakage per year. The incidence decreased after the introduction of the changes in clinical practice, and the highest value in period 2 was consistently lower than the lowest value in period 1.

### Logistic regression analysis

The logistic regression resulted in an unadjusted OR for anastomotic leakage of 0.43 (95% CI 0.25–0.73, *p* = 0.002) for all patients operated with colorectal resections in period 2 versus period 1 (Table 3). The crude effect of age, sex, type of resection, type of pathology, multivisceral resection, acute surgery and protecting ostomy on anastomotic leakage was statistically not significant. The multivariable logistic regression indicated no confounding effect of the predictors above, nor were their interaction terms significant. The model adjusted for age, sex, type of resection and acute surgery resulted in an OR of 0.45 (95% CI 0.26–0.78, *p* = 0.004). The multivariable analysis restricted to elective colorectal procedures yielded an OR of 0.47 (95% CI 0.27–0.82, *p* = 0.007) after adjustment for age, sex, type of resection and acute surgery.

**Table 3:** Non-adjusted logistic analysis of risk factors for anastomotic leakage

Variable	Odds ratio	95% confidence interval	*p* value
Men	1 (ref)		
Women	0.65	0.38–1.11	0.114
Age (years)	1.01	0.99–1.03	0.211
Pathology			
Cancer	1 (ref)		
IBD	0.81	0.36–1.81	0.603
Other	n.a.	n.a.	n.a.
Type of resection			
Colon	1 (ref)		
Rectal	1.21	0.72–2.03	0.482
No multivisceral resection	1 (ref)		
Multivisceral resection	1.50	0.89–2.53	0.128
Elective resection	1 (ref)		
Emergency resection	1.40	0.54–3.66	0.489
No protective ostomy	1 (ref)		
Protective ostomy	0.78	0.44–1.37	0.386
Period 1 (2006–2009)	1 (ref)		
Period 2 (2010–2013)	0.43	0.25–0.73	0.002

Period 1 and period 2 are referring to before and after introduction of changes in clinical practice

n.a. = not applicable due to no events

### Length of stay

The median length of stay for participants with an anastomotic leakage was longer; 25 (6–190) days versus 9 (2–167) days, (*p* < 0.001) in the entire study population. The median length of stay was shorter after the introduction of changes in clinical practice; 9 (3–119) days versus 10 (2–190) days (*p* = 0.043) (Table 4).

**Table 4:** Median length of stay for patients operated with colorectal resections in period 1 *versus* period 2, and for patients with *versus* without anastomotic leakage

Group	*n*	LOS	Range	*p*
Period 1	409	10	2–190	
Period 2	485	9	3–119	0.043
Leakage	63	25	6–190	
No leakage	831	9	2–167	<0.001

Median length of stay (LOS). *p* value Wilcoxon rank-sum test

## Discussion

This cohort study shows that the anastomotic leakage rate after colorectal resections with a primary anastomosis decreased from 10.0 to 4.5% after implementing a structured change in clinical practice including exclusion of perioperative NSAIDs, introduction of intra-operative goal-directed fluid therapy and avoidance of primary anastomoses in acute resections. However, the present study design cannot determine to what extent each component contributed to the reduction in anastomotic leakage rate.

Avoiding colorectal anastomoses in emergency procedures may prevent anastomotic leakage since emergency resection with primary anastomosis has been identified as an independent risk factor for anastomotic leakage [2]. The proportion of emergency resections was small in the present study which may explain why this effect was not detected, as analysis restricted to elective procedures showed similar findings as for the entire study population. Previous studies suggest that omission of perioperative NSAIDs and optimizing intra-operative fluid balance are factors that may reduce the risk of anastomotic leakage [9, 27]. Intra-operative fluid and sodium overload has been associated with increased hospital stay and complication rate, but can be prevented by restricted fluid therapy regimens [28–30]. Also hypovolemia may increase complications. Different methods for goal-directed fluid therapy in order to obtain better fluid balance have been described and seem to improve postoperative outcomes in terms of shorter length of stay and reduced complication rates [31], and has been recommended as part of ERPs [32]. Esophageal Doppler for intra-operative stroke volume optimization was introduced at our unit for colorectal surgery and may allow for better hemodynamic control, especially during longer and more complex surgery [33]. The combined effect of the factors above appeared to have an effect in the present study and can be adopted at relatively low costs. In contrast, the cost of grade III complications after colorectal operations has been estimated to a mean of more than 50,000 US Dollars [34]. Our results are in agreement with a recent smaller study that reported a reduction in anastomotic leakage rate from 9.8 to 4.2% after implementation of a very similar quality improvement program [35].

### Limitations

The majority of patients referred to the Karolinska University Hospital have advanced colorectal cancer or complex inflammatory bowel disease, which is reflected by a high proportion of simultaneous multivisceral resections. The risk of selection bias was minimized by inclusion of all consecutive patients undergoing colorectal resection with primary anastomosis. During the entire study period the referral pattern did not change and patients treated before and after the changes in clinical practice were comparable regarding age, sex, type of diagnosis, type of resection, multivisceral resection and protective ostomy. Principles of care, except from the described changes in clinical practice, and senior medical staff remained unchanged during the study period. An overestimation of NSAIDs use during period 1 and goal-directed fluid therapy during period 2 cannot be excluded but would bias the presented estimates toward no effect. Preoperative irradiation has been identified in some studies as an independent risk factor for anastomotic leakage after low anterior resection [7, 36], while other studies have not [37]. The present study is based on a local quality database without information about neo-adjuvant treatment. A majority of the patients operated with low anterior resection were operated for rectal cancer (85%). Approximately 75% of these patients, irrespectively of period, received preoperative radiotherapy according to the national Swedish colorectal cancer register. Increased attention of involved surgeons to the problem of anastomotic leakage, especially at the time of the introduction of changes in clinical practice, may have improved the results in period 2 but cannot be quantified. Preoperative intervention for smoking cessation seems to reduce the risk for postoperative complications, but the policy regarding smoking cessation did not change during the study period [38]. Age, sex, type of resection, type of pathology, multivisceral resection, acute surgery and protecting ostomy had no important confounding effect in this study. It is not likely that other patient-related risk factors for anastomotic leakage, not assessed in this study, changed between period 1 and 2 to an extent that would explain the difference in leakage rate.

A subgroup of asymptomatic grade A leakages, commonly associated with late leakage, were not registered in this trial. They represent a minority of leakages that do not alter the clinical course during the index hospitalization, but are relevant as they may prevent or delay the subsequent closure of a protective ostomy, or impair functional outcome after low anterior resection.

### Generalizability

The internal validity of the presented findings is robust as the limitations discussed above do not suggest that the observed reduction in anastomotic leakage can be attributed to other factors than the described changes in clinical practice. The external validity might be restricted to some extent as the study population had more multivisceral resections than the general population undergoing colorectal resections and the proportion of minimally invasive procedures was very small. However, from a global perspective open surgery is still the dominating surgical method.

## Conclusion

Implementing a structured change of clinical practice can significantly reduce the anastomotic leakage rate after colorectal resections.
